# Neurotrophic-tyrosine receptor kinase gene fusion in papillary thyroid cancer: A clinicogenomic biobank and record linkage study from Finland

**DOI:** 10.18632/oncotarget.28555

**Published:** 2024-02-05

**Authors:** Wei Zhang, Arndt A. Schmitz, Roosa E. Kallionpää, Merja Perälä, Niina Pitkänen, Mikko Tukiainen, Erika Alanne, Korinna Jöhrens, Renate Schulze-Rath, Bahman Farahmand, Jihong Zong

**Affiliations:** ^1^Bayer HealthCare Pharmaceuticals Inc, Whippany, NJ 07981, USA; ^2^Bayer AG, Berlin, Germany; ^3^Auria Biobank, Turku University Hospital, University of Turku, Turku, Finland; ^4^Department of Oncology and Radiotherapy, Turku University Hospital, Turku, Finland; ^5^Dresden University Hospital, Technical University Dresden, Dresden, Germany; ^6^Bayer AB, Solna, Sweden; ^7^Western Finland Cancer Centre, Turku, Finland

**Keywords:** NTRK gene fusion, papillary thyroid cancer, clinicogenomic, epidemiology, biobank

## Abstract

Selective tropomyosin receptor kinase (TRK) inhibitors are approved targeted therapies for patients with solid tumors harboring a neurotrophic tyrosine receptor kinase (*NTRK*) gene fusion. Country-specific estimates of *NTRK* gene fusion frequency, and knowledge on the characteristics of affected patients, are limited. We identified patients with histologically-confirmed papillary thyroid cancer (PTC) from Finland’s Auria Biobank. TRK protein expression was determined by pan-TRK immunohistochemistry. Immuno-stained tumor samples were scored by a certified pathologist. Gene fusions and other co-occurring gene alterations were identified by next generation sequencing. Patient characteristics and vital status were determined from linked hospital electronic health records (EHRs). Patients were followed from 1 year before PTC diagnosis until death. 6/389 (1.5%) PTC patients had an *NTRK* gene fusion (all *NTRK3*); mean age 43.8 years (and none had comorbidities) at PTC diagnosis. Gene fusion partners were *EML4* (*n* = 3), *ETV6* (*n* = 2), and *RBPMS* (*n* = 1). Of 3/6 patients with complete EHRs, all received radioactive iodine ablation only and were alive at end of follow-up (median observation, 9.12 years). In conclusion, *NTRK* gene fusion is infrequent in patients with PTC. Linkage of biobank samples to EHRs is feasible in describing the characteristics and outcomes of patients with PTC and potentially other cancer types.

## INTRODUCTION

Gene fusions involving members of the neurotrophic-tyrosine receptor kinase (*NTRK*) family are established oncogenic drivers across various adult and paediatric cancers [1]. Large real-world studies have reported an *NTRK* gene fusion prevalence of 0.3% among patients with solid tumors, although this varies by cancer type [2–5]. These fusions are commonly detected in rare cancers such as infantile (congenital) fibrosarcoma and secretory breast carcinoma, with prevalences over 90% [6, 7]. Conversely, they are rare in more common solid tumors such as lung adenocarcinoma (0.10–0.23%) [3, 4, 8], breast cancer (0.13–0.39%) [2, 3, 8], colorectal cancer (0.20–0.31%) [2, 4, 8], bladder cancer (0.34%) [2], and thyroid cancer (1.6–6.0%) [2–4, 8, 9].

The *NTRK* family includes three genes – *NTRK1*, *NTRK2* and *NTRK3* – encoding the transmembrane tropomyosin receptor kinase (TRK) A, B, and C proteins, respectively [10]. Fusion involving the 3’ kinase region of the *NTRK* gene with the 5’ region of its fusion partner gene (arising from intra-chromosomal or inter-chromosomal rearrangement) results in the formation of a chimeric protein with a kinase domain that is constitutively activated or overexpressed [11, 12]. This, in turn, causes downstream stimulation of cellular proliferation via the RAS/RAF/MAPK pathway [10].

Recent years have seen the landmark, tumor-agnostic regulatory approval of TRK inhibitors (larotrectinib and entrectinib) as treatment for patients with solid tumors harboring an *NTRK* gene fusion [1], with larotrectinib currently approved across 48 countries. These targeted therapies bind to the kinase domain inhibiting the rearranged chimeric oncoproteins, and have been associated with objective response rates of 71% (larotrectinib) [13] and 54% (entrectinib) [14] in clinical trials of patients with thyroid cancer harboring *NTRK* gene fusions. Genomic profiling of tumors to identify patients eligible for these drugs is not, however, routine in clinical practice. Estimates of the frequency of *NTRK* gene fusion in large cohorts of patients with solid tumors come from a limited number of studies. Furthermore, the challenge of recruiting affected patients for follow-up, when these fusions are largely seen in rare cancers, has led to a knowledge gap regarding prognosis, survival, and therapeutic outcome [10] with most current knowledge based on selected patients in clinical trials. We aimed to address this gap by evaluating patients with papillary thyroid cancer (PTC) from Finland using biobank stored samples as a source of genomic data linked to longitudinal hospital electronic health records (EHRs). To our knowledge, this has not been undertaken previously in the context of *NTRK* gene fusions. We selected PTC for this present study due to its relatively high prevalence of *NTRK* gene fusions compared with other cancer entities [2, 8], with a reported prevalence between 1.2% and 6.7% [10, 15–18], higher in paediatrics (19.2%–26.0%) [18, 19]. The study objectives were to determine the frequency of *NTRK* gene fusion in patients with PTC in Finland, and to describe co-occurring genetic alternations, clinical characteristics, disease progression, and treatment pathways in *NTRK* gene fusion positive patients.

## RESULTS

### Patients with *NTRK* gene fusion

A total of 6/389 (1.5%) of the PTC cohort tested positive for *NTRK* gene fusion. Genomic and other characteristics of these six patients are shown in Table 1; the vital status for all was confirmed by data from Statistics Finland. Three patients (numbers 1, 2 and 6) were diagnosed with PTC outside of the period 2005–2017, and therefore did not have complete EHR data and were not included in the subcohort. Consequently, the subcohort contained 3/234 patients positive for *NTRK* gene fusion (patients 3, 4, and 5 in Table 1). Four samples had tested positive upon immunohistochemistry (IHC) testing, with three confirmed as positive following NGS; no IHC negative samples were subsequently confirmed as positive (Table 2).

**Table 1:** Demographic and clinical characteristics of patients with PTC diagnosed between 1993 and 2018 and positive for *NTRK* gene fusion (*n* = 6)

	Patient 1^*^	Patient 2^*^	Patient 3	Patient 4	Patient 5	Patient 6^*^
**Demographics**						
Year of thyroid cancer diagnosis	1993	1993	2009	2009	2011	2018
Age group at cancer diagnosis (years)^†^	50–54	55–59	40–44	30–34	25–29	55–59
**Lifestyle factors/comorbidities**						
BMI (kg/m^2^)	N/A	N/A	22.4	25.8	22.6	22.98
Smoking status	N/A	N/A	Non-smoker	Current smoker	Non-smoker	Non-smoker
Comorbidities	N/A	N/A	None	None	None	None
CCI at thyroid cancer diagnosis	N/A	N/A	1	0	0	0
**Tumor characteristics**						
Stage at diagnosis (AJCC)	NA	NA	Unknown	Unknown	I	Unknown
Stage at diagnosis (TNM)	NA	NA	T1N0Mx	T1N1Mx	T1N1aM0	T1bNxMx
**Genetic characteristics**						
*NTRK* gene	*NTRK3*	*NTRK3*	*NTRK3*	*NTRK3*	*NTRK3*	*NTRK3*
*NTRK* gene fusion partner	*EML4*	*EML4*	*EML4*	*RBPMS*	*ETV6*	*ETV6*
Genomic co-alteration	None	None	None	*RB1* deletion	*NSG2::KIF5B*	*BRAF-V600E* *detected in the* *other lobe*
**Cancer treatments**						
Radiotherapy	N/A	N/A	No	No	No	No
Radioactive iodine ablation (RAI-131)	N/A	N/A	Yes	Yes	Yes	No
Time to first treatment (months)	N/A	N/A	13.5	4.0	2.6	N/A
Number of treatments	N/A	N/A	1	2	2	N/A
Chemotherapy	N/A	N/A	No	No	No	No
Observation length (years)	25.8	25.2	9.8	9.1	7.0	0.8
Vital status at end of follow-up^‡^	Alive	Alive	Alive	Alive	Alive	Alive

^*^Patient was diagnosed with PTC outside of the period 1995–2017 and therefore did not have EHR data. These three patients were not included in the subcohort. ^†^According to National Cancer institute Surveillance, Epidemiology, and End Results Program (NCI SEER data) age categorization. ^‡^Vital status on 30 November 2018. Note: Use of N/A is because these patients did not have EHR data. Abbreviations: AJCC: American Joint Committee on Cancer; BMI: body mass index; N/A: not applicable; *NTRK*: neurotrophic tyrosine receptor kinase; PTC: papillary thyroid cancer; RB1: RB transcriptional corepressor 1; TNM: tumor-node-metastasis; Mx: metastasis unknown.

**Table 2:** *NTRK* gene fusion results from IHC and NGS testing of samples from the subcohort (*N* = 234)

	NGS positive, *n*	NGS negative, *n*
IHC positive	3	1
IHC negative	0	228

Valid IHC data were available from 232 samples (from 223 patients) for which both NGS and IHC data on any sample were available. Abbreviations: IHC: immunohistochemistry; NGS: next generation sequencing.

Of the six patients positive for *NTRK* gene fusion in the study cohort, all tested positive for *NTRK3*. The STAR-Fusion analyses confirmed the presence of in-frame junction reads in the correct orientation in all cases. Also, in all cases, the *NTRK* kinase domain was preserved and the ‘left’ and ‘right’ breakpoints of the two genes were identified. Finally, in two of these six cases, reads corresponding to the reciprocal translocation were additionally identified. The gene fusion partner was *EML4* in three patients, *ETV6* in two patients and *RBPMS* in one patient. Of the two patients with a *ETV6* gene fusion partner, one contained an established PTC oncogenic *BRAF-V600E* mutation (note, for this patient, *ETV6::NTRK3 fusion* was determined from a sample from one lobe of the thyroid gland, while the BRAF mutation was determined from a separate sample obtained from the other lobe of the thyroid gland a month later). The other patent with an *ETV6* gene fusion partner also exhibited a *NSG2::KIF5B* fusion of unclear clinical relevance. The patient with the *RBPMS* gene fusion partner also harbored a *RB1* deletion as a genomic co-alteration. Among the six patients positive for *NTRK* gene fusion, 50% were female, two were in the 55–59 age group at cancer diagnosis, and one each were in the following in age groups: 50–54 years, 40–44 years, 30–34 years, and 25–29 years. Cancer stage was known for one patient who had early-stage disease. Comorbidities at PTC diagnosis were not documented for any patient.

### Performance of the IHC assay

Because a large number of subcohort member samples were assessed by both NGS and IHC (Table 2), we were able to evaluate the analytical performance of the latter, taking the NGS findings as the gold standard [20]. Using the best performance criterium “tumor cytoplasm score >1” as the threshold gave a sensitivity of 100.00%, a specificity of 99.57%, a positive predictive value of 75%, a negative predictive value of 100%, and an accuracy of 99.57%. The cytoplasm was the subcellular tumor compartment predominantly stained (mostly weakly). No sample was scored as category ‘3’, and no sample showed perinuclear tumor cell pan-TRK staining. Two samples showed nuclear staining and both samples were NGS positive. However, while the overall accuracy of such a threshold definition would be the same (data not shown), its sensitivity would be lower because one of three NGS positive cases would not be detected. Thus, the threshold defined above was preferred from a clinical perspective. NGS testing also revealed that the two cases with nuclear staining were those where *NTRK3* was fused to the transcription factor *ETV6* (Patient 5) and to *RBPMS* (Patient 4) involved in maturation of mRNA, respectively. The case without nuclear staining was the patient where *NTRK3* was fused to *EML4* (Patient 3), which is located in the nucleus and encodes a protein associated with the mitotic spindle. Thus, as also noted by others [8], the biological role of the fusion partner can influence the subcellular localization of the *NTRK* fusion chimeric protein.

### Characteristics of the subcohort

Of the 234 patients in the subcohort, just over a quarter were male (26%), mean age at thyroid cancer diagnosis was 53 years, and median Charlson Comorbidity Index (CCI) score at diagnosis was 2.0. Characteristics stratified by *NTRK* gene fusion status (positive/wild type) are shown in Table 3; however, comparisons between the two groups were limited based on there being only three patients in the *NTRK* fusion positive group. Among patients with *NTRK* wildtype, 69% were treated with radioactive iodine ablation, 16% with radiotherapy and 12% with chemotherapy, and 86% were still alive at the end of follow-up (median 7.2 years’ observation). Among the three patients in the *NTRK* fusion positive group, all three (100%) received radioactive iodine ablation (none received chemotherapy, radiotherapy or a TRK inhibitor), and all were alive at the end of follow-up (median 9.12 years’ observation, range 8–10 years).

**Table 3:** Characteristics of the 234 patient subcohort diagnosed from 2005–2017 with PTC and with *NTRK* gene fusion data and linked EHR data

Characteristic	NTRK gene fusion *N* = 3 *n* (%)	NTRK wild type *N* = 231 *n* (%)
**Age at thyroid cancer diagnosis**		
Median (IQR)	31.0 (15.0)	56.0 (23.0)
18–39	2 (66.7)	51 (22.1)
50–54	1 (33.3)	60 (26.0)
≥55	0 (0)	120 (52.0)
**Sex**		
Female	1 (33.3)	173 (74.9)
Male	2 (66.7)	58 (25.1)
**BMI, kg/m2**		
<30 (non-obese)	3 (100.0)	117 (50.7)
≥30 (obese)	0 (0.0)	42 (18.2)
Missing	0 (0.0)	72 (31.2)
**Smoking status**		
Current	1 (33.3)	45 (19.5)
Former	0 (0.00)	35 (15.15)
Never	2 (66.7)	117 (50.7)
Missing	0 (0.0)	34 (14.7)
**Charlson Comorbidity Index at diagnosis**		
Median (IQR)	0.0 (1.0)	2.0 (3.0)
0–2	3 (100)	116 (50.2)
3–4	0 (0.0)	81 (35.1)
≥5	0 (0.0)	34 (14.7)
Cancer stage at diagnosis, AJCC^*^		
I	1 (33.3)	69 (29.9)
II	0 (0.0)	9 (3.9)
III	0 (0.0)	2 (0.9)
IV	0 (0.0)	2 (0.9)
Unknown	2 (66.7)	149 (64.5)
**Cancer treatments**		
Radiotherapy	0 (0.0)	36 (15.6)
Radioactive iodine ablation	3 (100.0)	159 (68.8)
Chemotherapy	0 (0.0)	28 (12.1)
Procedures^*^, median (IQR)		
Total	46 (29.0)	33 (39.0)
Before PTC diagnosis	6 (3.0)	6 (16.0)
After PTC diagnosis	40 (32.0)	25 (26.0)
**Hospitalizations (mean number per patient)**		
Visit	215 (71.7)	16,669 (72.2)
Ward	14 (4.7)	1447 (6.3)
**Deaths/survival**		
Length of observation while alive (median, IQR)	9.12 (2.8)	7.23 (5.3)
Total deaths	0 (0.0)	32 (13.9)
Deaths within 5 years	0 (0.0)	16 (6.9)
Death within 10 years	0 (0.0)	30 (13.0)

^*^Included extensive surgical operations, small operations, medical imaging, device-assisted examinations and some therapies. ^*^AJCC stage was derived based on the available TNM data. Unknown classification is due to missing or incomplete TNM. Abbreviations: AJCC: American Joint Committee on Cancer; BMI: body mass index; EHR: electronic health record; IQR: inter-quartile range; *NTRK*: neurotrophic tyrosine receptor kinase.

## DISCUSSION

In this population-based study among patients diagnosed with PTC and with an archived PTC tissue sample in the Auria Biobank, the frequency of *NTRK* gene fusion was 1.5% (6/389). All six patients with an *NTRK* gene fusion tested positive for *NTRK3*, and the most common gene fusion partner was *EML4* (three patients), followed by *ETV6* (two patients) and *RBPMS* (one patient). We have also demonstrated the ability of biobank–EHR linkage for longitudinal follow-up of these patients enabling their characterization, and evaluation of treatment patterns and survival.

Our study comprising 389 patients and 456 tissue samples provided substantial coverage of patients with thyroid cancers in the Hospital District of Southwest Finland 1993–2018 because there were 722 new thyroid cancer diagnoses in this region during this period [21]. The observed 1.5% frequency of *NTRK* gene fusion is similar to the prevalence seen in the US Cancer Genome Atlas project [15], where 1.2% (6/484) of patients with PTC (excluding clinically aggressive tumors) harbored an *NTRK* gene fusion. Both of these estimates are slightly lower than those reported in other large cohorts of patients with PTC from the Czech Republic (6.7%, 57/846) [10], Taiwan (23.%, 12/525) [17], China (3.3%, 12/355) [16], and the Middle East (6.0%, 9/315) [18]. Frequencies of *NTRK* gene fusions from smaller cohorts have similarly varied by geographical region with estimates of 12.1% (4/33) in Poland [22], 11.8% (9/76) in Italy [23], 5.3% (1/19) in France [24], and 0% (0/14) in China [25]. All six patients positive for *NTRK* gene fusion in our study harbored rearrangement of the *NTRK3* gene, which has been found to be the most frequent *NTRK* gene fusion in other PTC cohorts [9, 10, 15, 17, 18, 26]. *NTRK1* gene fusions are also established in thyroid cancer, albeit less commonly than *NTRK3* gene fusions [3, 10]; for example, Pekova et al. [10, 16] found that *NTRK3* gene fusions were five times more common than *NTRK1* gene fusions in their cohort of 846 patients with PTC. No patient in our study cohort had a *NTRK2* gene fusion, consistent with the lack of reported cases in thyroid cancer in the literature. The *NTRK3* fusion partners found in our study – *EML4, ETV6*, and *RBPMS* – have all been previously documented in the literature [10, 15–17, 19, 26, 27], especially *ETV6::NTRK3* gene fusions, seemingly the most common detected *NTRK* gene fusion detected in other PTC cohorts [10, 15, 26]. Other *NTRK* gene fusions reported in other studies include *SQSTM1::NTRK3* [10, 16, 26], *ERC1::NTRK3* [17], *IRF2BP2::NTRK1* [10, 16], *TPR::NTRK1* [10, 19, 26], *TPM3::NTRK1* [10], and *SQSTM1::NTRK1* [10, 26]. Interestingly, one of the two patients in our cohort with a *ETV6::NTRK3* gene fusion had a *BRAF-V600E* genomic alteration detected from a tissue sample originating from the other lobe of the thyroid gland than the sample from which the *ETV6::NTRK3* fusions was determined. Pekova et al. [10] similarly describes a case where a patient with multifocal PTC harboring an *ETV6::NTRK3* gene fusion in a nodule in the right thyroid lobe had a *BRAF-V600E* mutation detected in a nodule in the left thyroid lobe. While intratumor heterogeneity is a well-known phenomenon [28, 29], it is important to note there was no co-occurrence of the two oncogenes *NTRK* gene fusion and *BRAF* mutation in the same lobe of the thyroid gland.

Following the introduction of TRK inhibitors for the treatment of patients with solid tumors harboring an *NTRK* gene fusion, observational cohorts of patients receiving these drugs are required to evaluate treatment outcomes in real-world settings. None of the six patients with PTC positive for *NTRK* gene fusion in the cohort received TRK inhibitor therapy because they did not have metastatic disease; additionally, these drugs were not approved in Finland at that time. Outside of clinical trials, the effectiveness of larotrectinib in patients with PTC harboring a *NTRK* gene fusion has thus far been demonstrated in case studies [30–32]. Our present study demonstrates the ability to create a clinicogenomic dataset through harnessing biobank sample derived genomic data, linked EHRs and vital status records, and to describe and follow-up such patients to evaluate natural history, real-world treatment outcomes, and prognosis. It shows the potential to conduct larger studies of this kind in future involving the evaluation of real-world TRK treatment outcomes. These could be among larger cohorts of patients with PTC (through ongoing identification of more *NTRK* fusion patients) as well as potentially among patients with other cancer types. The median follow-up of the three patients with an *NTRK* gene fusion in the subcohort at the time of the analysis was over 9 years, which indicates a good length of longitudinal data to observe long-term outcomes, treatment patterns and prognosis. However, as the availability of EHRs (rather than paper-based from records) were largely available from 2005 onwards, this led to a smaller subcohort being described.

Strengths of this study are the population-based sample representative of the region of Finland from which it was drawn meaning our findings have good generalizability, and the long follow-up. The information included in the data sources enabled the description of a wide range of patient characteristics including tumor features and treatments received. Turku University Hospital covers cancer care in the region, and the hospital EHR database includes both inpatient and outpatient data, thus the level of data completeness on disease stage and cancer treatments was high in the subcohort. Another strength is the high sensitivity (100%) and specificity (99.57%) of the pan-TRK IHC for detecting *NTRK* gene fusions, showing that the IHC was able to detect the same number as fusions as NGS, and indicating that *NTRK* gene fusion misclassification was likely to be minimal. In a previous study that included 571 patients with thyroid cancer, pan-TRK IHC demonstrated 81.8% sensitivity and 100% specificity for the 38 cases where IHC was undertaken [8]. Additionally, sensitivities of 75–95% and specificities of 82–100% for pan-TRK IHC across various tumor types have been reported in the literature [4, 8, 33, 34]. Further, to the best of our knowledge, our subcohort is the largest thyroid cancer cohort yet described where all FFPE samples underwent pan-TRK IHC and NGS confirmation. However, clinical adoption of IHC requires setup of the assay and availability of an experienced pathologist. The main weakness of the study was that the small number of patients testing positive for *NTRK* gene fusion prevented the identification of other potential gene fusion partners and limited meaningful comparisons with wild-type *NTRK* patients.

In conclusion, this study showed the frequency of *NTRK* gene fusions in patients with PTC to be 1.5%. Furthermore, the creation of a clinicogenomic dataset based on genomic data from the Auria biobank linked to longitudinal EHRs and vital statistics is a feasible method of conducting a real-world observational study of patients with PTC, and potentially those with other cancer types, who harbor an *NTRK* gene fusion.

## MATERIALS AND METHODS

### Study design and data source

This was a retrospective cohort study from Finland using tumor genomic data from patients with PTC held in the Auria Biobank linked to hospital EHR and vital status records. These data sources have previously been successful in determining biomarker expression among patients with malignant mesothelioma, and head and neck squamous cell carcinoma, as well as in evaluating their characteristics, prognosis and survival [35, 36]. The Auria Biobank is situated in the Turku region of Finland and was founded as a joint institution between the University of Turku and the hospital districts of Southwest Finland, Satakunta and Vaasa [37]. At the end of 2018, the population in the area of Hospital District of Southwest Finland was 481,478, with an average age of 42.2 years for males and 44.9 years for females, and with the majority being of Finnish descent [38]. Patients with cancer in this population are primarily treated at Turku University Hospital.

Launched in 2014, the Auria Biobank stores human biological samples collected for future health/medical research with the donor’s consent. In addition, the biobank stores diagnostic tissue samples that were collected before 1 September 2013 and legally transferred from hospital pathology collections (based on a public announcement procedure with a statement that the samples and information may be used for biobank research, according to Finnish law). These samples are linkable at the individual patient level to hospital EHRs in Turku University Hospital, which contain information on a wide range of patient and tumor characteristics, as described in Supplementary Table 1. Further, the hospital vital status records have high validity and inform the national Statistics Finland vital statistics database. Minimal migration that occurs out of the Turku region, combined with further linkage to the Statistics Finland national vital database, provides the potential for longitudinal observation of patients with minimal loss to follow-up. For this present study, use of the thyroid cancer tumor samples, and other patient data, were approved by Auria Biobank’s Scientific Steering Committee under Decisions AB18-6900 and AB18-9957, Hospital District of Southwest Finland (research permission T278/2018) and by Statistics Finland (research permission TK-53-448-20).

### Study cohort and subcohort

**Figure 1:**
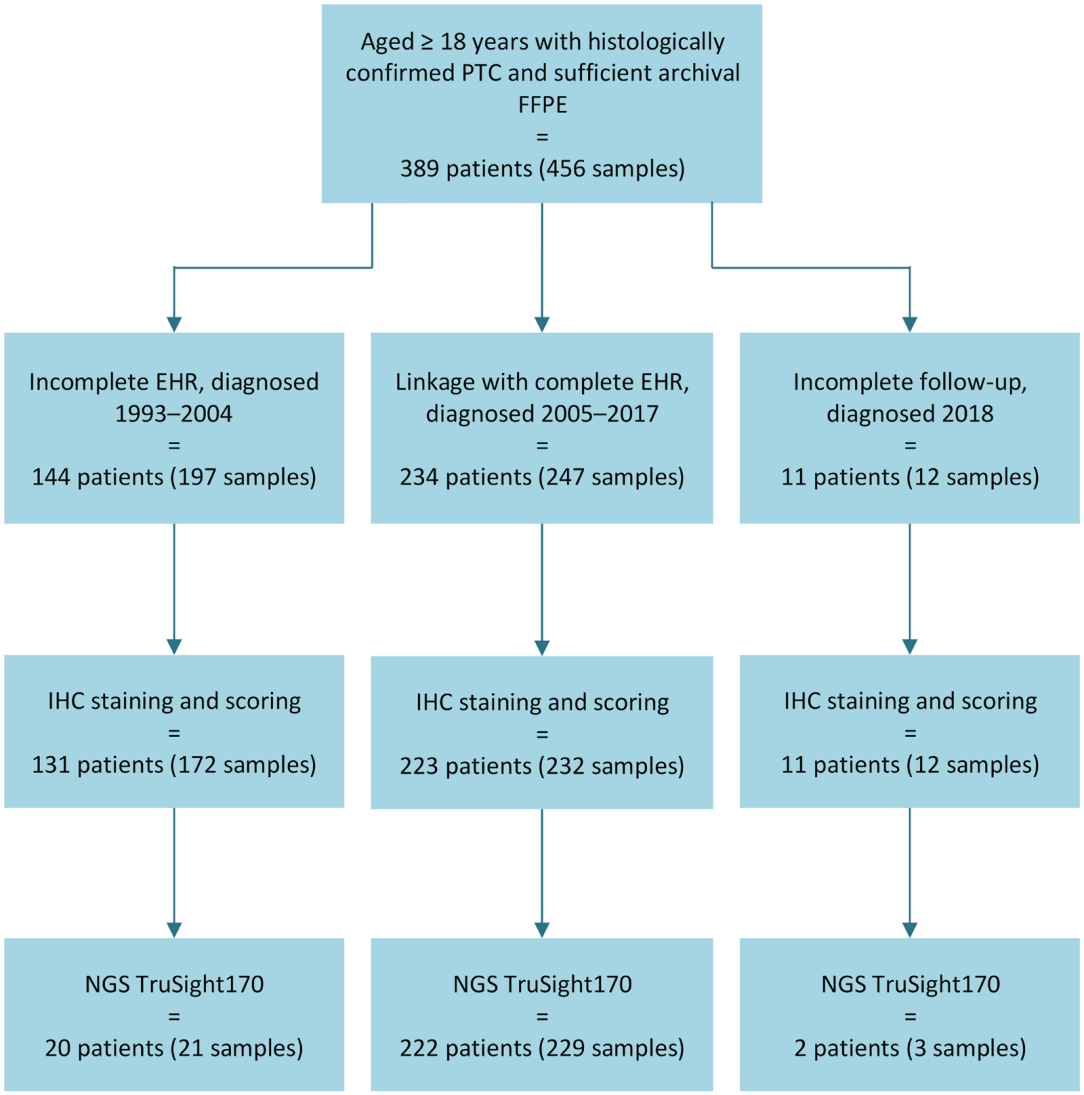
Flowchart depicting patient selection. Abbreviations: EHR: electronic health record; FFPE: formalin-fixed paraffin-embedded; IHC: immunohistochemistry; NGS: next generation sequencing; PTC: papillary thyroid cancer.

Identification of the PTC study cohort and subcohort is depicted in Figure 1. We included all individuals aged ≥18 years with a histologically confirmed diagnosis of PTC in the database of the Department of Pathology, Turku University Hospital (between 1993 and mid-2018) who also had at least one formalin-fixed paraffin-embedded (FFPE) tumor sample available and sufficient for research in the Auria Biobank (a total of 456 FFPE tumor samples from 389 patients). Subsequently, we identified a subcohort of 234 patients whose PTC was diagnosed between 2005 (when the hospital EHR data became most complete) and 2017. Of the 155/389 patients not included in the subcohort, the majority (144 patients) were diagnosed between 1993 and 2004 and so had an incomplete EHR, and 11 patients had incomplete follow-up due to being diagnosed during 2018.

### *NTRK* gene fusion identification

TRK protein expression was determined by pan-TRK IHC, and presence of *NTRK* gene fusions was confirmed by next generation sequencing (NGS) (Figure 1).

### Pan-TRK IHC

Automated IHC was performed on 5 μm histological FFPE sections on a Ventana Discovery autostainer using pan-TRK antibody (clone EPR17341) from Abcam (Cambridge, MA, USA) with DAB detection chemistry as described previously [34]. Positive and negative FFPE samples were used as positive and negative staining run controls. Stained slides were scored by a board-certified pathologist from Dresden University Hospital (KJ) within weeks after staining using the original glass slides. A sample for one patient that could not be scored was excluded (because the tissue was detached from the slide), and a further 39 samples (from 23 patients) that showed no tumor content on the IHC stained slide were also excluded. For each of the remaining 416 samples (from 365 patients), we estimated the percentage of tumor and normal adjacent cells on each slide. Staining was scored in four different categories: ‘0’ for no pan-TRK staining, ‘1’ for weak, ‘2’ for moderate, and ‘3’ for strong. For the normal adjacent area, the percentage of area with the highest score was estimated (e.g., if 20% of the normal adjacent area was scored 1, this implied that 80% was scored 0). The same was undertaken for the tumor area, which was further divided by subcellular compartment (cytoplasmic, membranous, perinuclear, and nuclear). All tumor cells, and not only hot spots, were evaluated.

### NGS confirmation

All samples (except one being too scarce) from subcohort members were successfully stained, scored, and underwent NGS confirmation. For members of the study cohort not included in the subcohort (i.e., those without linked EHR data), the samples most strongly stained in the tumor area were selected for NGS confirmation. Next generation sequencing was performed using the TruSight Tumor 170 (TST170) Kit (Illumina, San Diego, CA, USA), which was chosen for its wide coverage across 170 genes associated with solid tumors, detecting single-nucleotide variants/indels, and amplifications from DNA, as well as fusions/splice variants on a subset of genes (including *NTRK1/2/3*) from RNA. The complete panel of tested genes and content can be found elsewhere [39]. For confirmation of positive samples from pan-TRK IHC and determination of the fusion partner, as well as detection of co-occurring changes in oncogenes, samples nominated for NGS TST170 were analyzed on a Nextseq 500 system at the EN ISO 15189 accredited laboratory Biopticka S.R.O. (Plzeň, Czech Republic). Fusion calling was undertaken using Illumina’s algorithm V2.0.1.8, and correctness of the algorithm was confirmed by concordance with STAR-Fusion [40].

### Linkage to EHR and follow-up

We collected information from the hospital EHR database on demographics (age at thyroid cancer diagnosis and sex), comorbidities at cancer diagnosis using the modified Charlson comorbidity diagnosis list [41], CCI, lifestyle variables (body mass index (BMI) and smoking status), cancer treatments (e.g., chemotherapy, radiotherapy), and number of surgeries/hospitalisations (see Supplementary Table 1 for more details). Subcohort members were followed from 1 year before the date of thyroid cancer diagnosis until the end of follow-up, death, or the end of the study.

### Statistical analysis

Data analysis was exploratory and descriptive in nature with no pre-specified hypotheses. Frequency of *NTRK* gene fusion was expressed as a percentage of the total cohort who were confirmed positive for *NTRK* gene fusion. Characteristics of the subcohort were described using frequency counts and percentages for categorical variables, and medians with interquartile range (IQR) for continuous variables. Descriptions were performed separately for patients positive for *NTRK* gene fusion and those with wild-type *NTRK*. Analyses were performed using SAS version 9.4.

## SUPPLEMENTARY MATERIALS



## Abbreviations

BMI: body mass index
BRAF: proto-oncogene B-Raf
CCI: Charlson Comorbidity Index
EHR: electronic health records
EML: echinoderm microtubule-associated protein-like
ETV: ETS variant transcription factor
KIF: kinesin family
IHC: immunohistochemistry
IQR: inter-quartile range
MAPK: mitogen activated protein kinase
mRNA: messenger ribonucleic acid
NGS: next generation sequencing
NTRK: neurotrophic tyrosine receptor kinase
PTC: papillary thyroid cancer
RAF: rapidly accelerated fibrosarcoma
RAS: rat sarcoma
RB: retinoblastoma tumor suppressor
RBPMS: RNA binding protein with multiple splicing
TRK: tropomyosin receptor kinase
