# Supplementary material for: Neurotrophic-tyrosine receptor kinase gene fusion in papillary thyroid cancer: A clinicogenomic biobank and record linkage study from Finland
Source: Oncotarget. 2024 Feb 5;15:106–16. doi: 10.18632/oncotarget.28555 (PMC10852057; doi:10.18632/oncotarget.28555)
Supplement: Supplementary file 1 [file oncotarget-15-28555-s001.pdf]

Neurotrophic-tyrosine receptor kinase gene fusion in papillary thyroid cancer: A clinicogenomic biobank and record linkage study from finland

SUPPLEMENTARY MATERIALS

Supplementary Table 1: Type and format of information available in Turku university hospital EHRs

Dataset	Structured data	Unstructured data
Demographics	Date of birth<br>Sex	N/A
Tumor characteristics	Date of diagnosis<br>Sample collection date<br>Stage at diagnosis<br>ICD-10/ SNOMED T-/N-/M*	Tumor size<br>Date of metastasis<br>Metastasis location
Comorbidities	Date of diagnosis<br>Diagnosis code ICD-10	N/A
Procedures	Date of procedure Procedure code/description	N/A
Anticancer drugs	Date of administration<br>Agent<br>Route of administration<br>Dose	Treatment/course intent<br>Treatment/clinical response
Radiotherapy	Date of administration<br>Target<br>Course intent<br>Dose (first, last fractions)	Treatment/clinical response
Drugs	Date of administration<br>ATC code<br>Route of administration	N/A
Laboratory tests	Date of test<br>Test type<br>Test result	N/A
Hospitalizations	Date of hospitalization (start, end)<br>Ward type	Discharge notes
Lifestyle, risk factors	None	Height<br>Weight<br>Smoking status
Survival/observation time	Date of death<br>Date of last contact	N/A

*SNOMED II classification of the pathology samples (Systematized Nomenclature of Medicine version II). Abbreviations: T: topography; M: morphology; None: no information was available; N/A: not applicable as additional information was not explored.
